# A Case Report and Genetic Characterization of a Massive Acinic Cell Carcinoma of the Parotid with Delayed Distant Metastases

**DOI:** 10.1155/2013/270362

**Published:** 2013-04-03

**Authors:** Anthony C. Nichols, Michelle Chan-Seng-Yue, John Yoo, Sumit K. Agrawal, Maud H. W. Starmans, Daryl Waggott, Nicholas J. Harding, Samuel A. Dowthwaite, David A. Palma, Kevin Fung, Bret Wehrli, S. Danielle MacNeil, Philippe Lambin, Eric Winquist, James Koropatnick, Joe S. Mymryk, Paul C. Boutros, John W. Barrett

**Affiliations:** ^1^Department of Otolaryngology Head and Neck Surgery, The University of Western Ontario, Victoria Hospital, London Health Science Centre, Room B3-431A, 800 Commissioners Road East, London, ON, Canada N6A 5W9; ^2^London Regional Cancer Program, London, ON, Canada N6A 4L6; ^3^Lawson Health Research Institute, London, ON, Canada N6C 2R5; ^4^Department of Oncology, The University of Western Ontario, London, ON, Canada N6A 4L6; ^5^Department of Pathology, The University of Western Ontario, London, ON, Canada N6A 5C1; ^6^Informatics and Biocomputing Platform, Ontario Institute for Cancer Research, Toronto, ON, Canada M5G 1L7; ^7^Department of Radiation Oncology (Maastro), GROW School for Oncology and Developmental Biology, Maastricht University Medical Centre, Maastricht, The Netherlands; ^8^Department of Medical Biophysics, University of Toronto, Toronto, ON, Canada M5G 2M9; ^9^Department of Pharmacology and Toxicology, University of Toronto, Toronto, ON, Canada M5S 1A8

## Abstract

We describe the presentation, management, and clinical outcome of a massive acinic cell carcinoma of the parotid gland. The primary tumor and blood underwent exome sequencing which revealed deletions in CDKN2A as well as PPP1R13B, which induces p53. A damaging nonsynonymous mutation was noted in EP300, a histone acetylase which plays a role in cellular proliferation. This study provides the first insights into the genetic underpinnings of this cancer. Future large-scale efforts will be necessary to define the mutational landscape of salivary gland malignancies to identify therapeutic targets and biomarkers of treatment failure.

## 1. Introduction

Salivary gland cancers account for 0.3–0.9% of all cancers [1, 2], and acinic cell carcinoma (AciCC) accounts for 5–11% of these [3, 4]. AciCC most commonly arises in the parotid gland and typically presents at an early stage allowing surgical treatment with favorable five-year survival rates in excess of 90% [4]. However, approximately 19% of cases present with advanced stage disease, which is associated with a higher rate of distant metastases and poorer survival [4]. To date, this tumor type has not been genetically characterized.

## 2. Case Report

**Figure 1:**
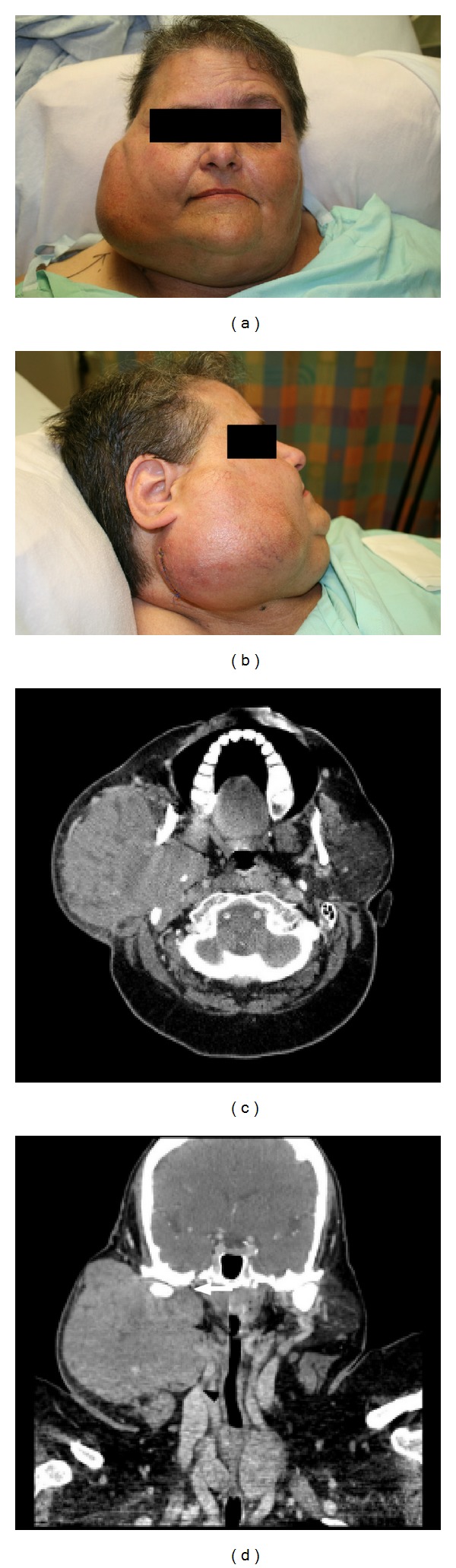
Anterior (a) and lateral (b) pictures of the patient at presentation. Axial (c) and coronal (d) CT scan images of the primary tumor. Note extension of the tumor into the parapharyngeal space with occlusion of the jugular vein (black arrowhead) and skull base involvement (white arrowhead).

**Figure 2:**
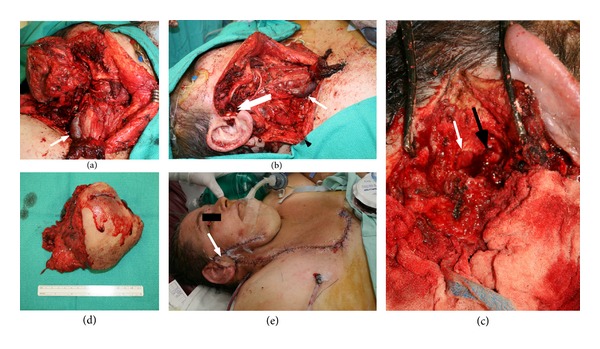
Intraoperative photographs demonstrating the radical parotidectomy with facial nerve sacrifice (a and b). (a) The large arrowhead indicates the divided jugular vein. (b) The single arrowhead (white) again marks the divided jugular vein, the double arrowhead (white) indicates residual tumor at the jugular foramen, and the black arrowhead indicates the divided end of the accessory nerve that was grafted to a cervical rootlet. (c) Temporal bone resection with surgicel occluding the sigmoid sinus (white arrowhead) and a fascia and muscle plug occluding the eustachian tube (black arrowhead). Demonstration of the primary tumor (d) and reconstruction with a large cervicofacial advancement flap and radial forearm free flap ((e) arrow marks forearm flap skin paddle).

**Figure 3:**
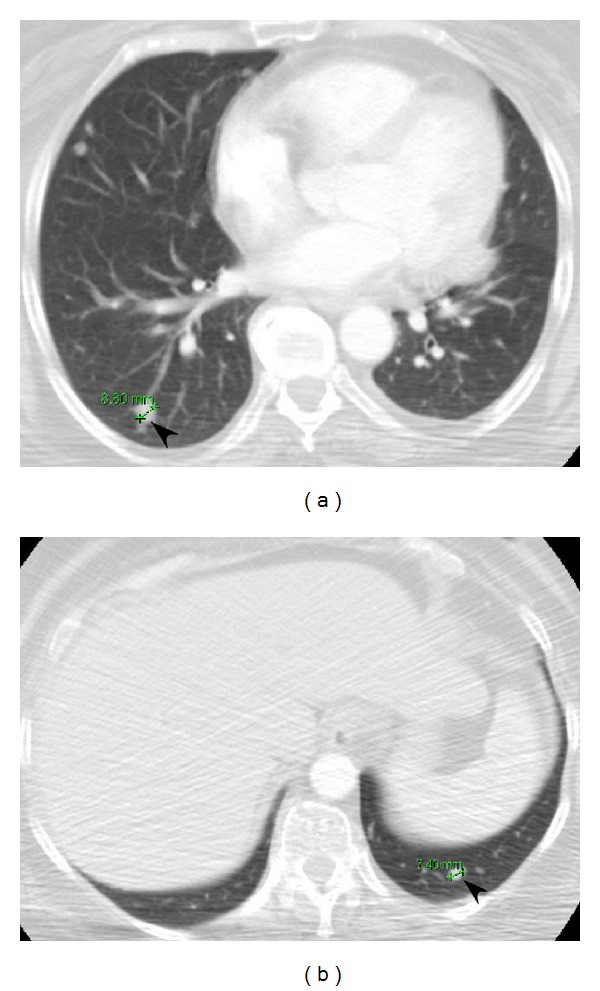
Axial CT scan images of the thorax (panels (a) and (b)) demonstrating the interval development of pulmonary metastases (arrowheads).

Our patient is a morbidly obese (375 lbs) 58-year-old woman who presented to the head and neck surgery clinic with a right parotid mass and intact facial nerve function. She underwent a fine needle aspiration which was consistent with a Warthin's tumor. CT imaging demonstrated a 5.8 cm right parotid mass. She was scheduled for surgery, but was lost to followup. She presented again to the head and neck surgery clinic one year later with significant interval growth of the mass and a partial right facial paresis (Figures 1(a) and 1(b)). A CT scan demonstrated a 11.2 × 9.4 × 10.7 cm mass centered in the right parotid effacing the jugular vein and abutting the mandible and skull base with extension along the facial nerve to the geniculate ganglion (Figures 1(c) and 1(d)). She was taken to the operating room and underwent a radical parotidectomy with facial nerve sacrifice, radical neck dissection, parapharyngeal space resection, and lateral temporal bone resection (Figures 2(a), 2(b), and 2(c)). The tumor was found to be invading the jugular foramen requiring occlusion of the sigmoid sinus and packing of the jugular foramen for vascular control. The tumor was also noted to be extending medially to the geniculate ganglion of the facial nerve. Gross tumor removal was accomplished (Figure 2(d)). We were not able to obtain a negative margin on the proximal facial nerve; thus, the ipsilateral masseter nerve was grafted to the buccal and marginal mandibular branches of the distal facial nerve. Her face was further rehabilitated with a static palmaris longus sling and temporary tarsorraphy, which was later replaced with a gold weight. The defect was reconstructed with a large cervicofacial rotation flap and a radial forearm free flap (Figure 2(e)). Pathologic examination revealed a 14 cm low-grade acinic cell carcinoma with extensive perineural and lymphovascular spread. The lymph node dissection yielded 29 lymph nodes, all of which were negative for malignancy. Postoperatively, she received 6000 cGy in 30 fractions using intensity modulated radiation therapy (IMRT) to the primary site and neck. During and after radiation, the patient experienced massive weight loss, losing approximately 200 lbs. Follow-up imaging one year after treatment revealed no evidence of local or regional recurrence; however, there was interval development of multiple new bilateral lung nodules up to 0.9 cm highly suspicious for metastases (Figures 3(a) and 3(b)). They were deemed too small to be biopsied percutaneously. The patient was referred for consideration of palliative chemotherapy; however, as she was asymptomatic the decision was made to follow her with serial imaging.

## 3. DNA Extraction, Exome Sequencing, and Bioinformatics Methods

Ethical approval was obtained from the University of Western Ontario Health Sciences Research Ethics and informed consent was obtained from the patient. DNA extraction from blood and tumor samples was carried out as previously described [5]. Exome sequencing was performed by Otogenetics (Tucker, Georgia) using the Agilent Human All Exon 50 Mbp exome capture kit with 30-fold coverage with 100 base-pair paired-end reads. The reference blood and primary tumor samples were aligned to the human hg19 reference sequence using Novoalign (v2.07.14). A maximum of five repeat alignments, defined as having a score difference of zero, were reported in the final output. SAM formatted output was specified with appropriate read group information provided. The remaining parameters were set to default values. Low-quality alignments, defined as alignments with low confidence in the reported position due to multiple alignment hits or poor base quality, were removed from the BAM files using SAMtools (v0.1.18) [6] by specifying the -q 30 quality filter. Additionally, unaligned and nonprimary reads (only 1 alignment, called the primary alignment, was retained in cases of multiple alignments) were removed by again using SAMtools (v0.1.18) and specifying the -F4 and –F 256 flags, respectively [6]. PCR artifacts were removed using MarkDuplicates tool from Picard (v1.66) with default settings. Samples were then processed as a matched set through the GATK (v1.3-16) pipeline [7, 8]. Samples were initially locally realigned using the IndelRealigner walker from the GATK package with known insertions and deletions found in dbSNP build 135. This was followed by base quality recalibration using GATK. Finally, variants were called and filtered using the GATK UnifiedGenotyper and VariantFiltration walkers again with default settings. Somatic variants within the targeted regions were identified using an in-house Perl library. To be classified as a somatic variant, the following conditions had to be met: (1) a tumor variant was identified by GATK and had a minimum 20x coverage and (2) the variant base was not seen at that position in the corresponding normal sample (20x coverage). The genes were annotated using RefGene and the consequences of the variations were identified using ANNOVAR dated 2012-03-08 [9]. As a final filtration step, any somatic variants, found in genes identified by Fuentes et al. as possible problematic genes for sequencing data, were removed [10]. Next, copy-number variants in the target regions were predicted with contra (v2.0.3) using default parameters [11]. DNAcopy (v1.32.0) was used to segment the copy numbers for visualization. This was done using default settings. Visualizations were generated using R (v2.15.2) and the lattice (v0.20-11) and latticeExtra (v0.6-24) packages. Of the target bases, approximately 60% were covered at a minimum of 20x collapsed coverage in the blood sample and 63% in the tumor.

## 4. Interpretation of Identified Mutations

**Figure 4:**
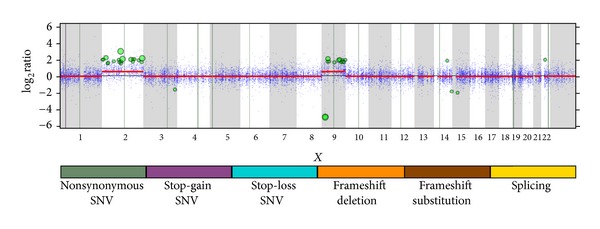
Somatic whole exome sequencing results across the genome. Vertical lines represent the single-nucleotide variants, while the points represent copy-number variant calls. Significant CNVs are represented by the green dots with its size reflecting its significance. Segmented copy-number variants are depicted in red and show clear gains in chromosome 2 and chromosome 9.

Our bioinformatics pipeline identified 14 nonsynonymous mutations (Table 1) and significant copy-number variations (CNVs) in 35 genes including 31 amplifications and 4 deletions (Table 2). CNVs were classified as significant if they met an adjusted *P* value threshold value of 0.05. A comprehensive listing of the single-nucleotide variants, (SNVs) identified including noncoding regions is provided in the Supplementary Table  S1 available online at http://dx.doi.org/10.1155/2013/270362. The amplifications occurred preferentially in chromosome 2 (*P* = 2.91 × 10^−21^) and chromosome 9 (*P* = 1.59 × 10^−18^), with single-amplification loci in chromosomes 14 and 22 (Table 2). The coding SNVs and all CNVs are illustrated in Figure 4. In addition, there were two deletions on chromosome 9 (CDKN2A and MTAP), one on chromosome 14 (PPP1R13B) and one on chromosome 3 (ETV5). All of the identified single-nucleotide variant mutations were heterozygous. Thirteen exhibited nonsynonymous changes in the respective proteins and one was a nonsense mutation which led to premature termination of the protein (GRIK3).

Several of these aberrations are plausibly associated to tumor formation and growth. Most critically, somatic deletion of the potent tumor-suppressor CDKN2A was identified. CDKN2A is one of the most widely mutated genes in human malignancies. According to the ICGC data-coordinating centre, it is mutated in up to half of glioblastoma multiforme, 14% of squamous cell carcinomas of the lung, and a third of all pancreatic adenocarcinomas [12]. It functions by stabilizing TP53 by sequestering the MDM2 ubiquitin ligase and by inhibiting CDK4-mediated G1 progression through the cellcycle. In addition, a deletion of 120 nucleotides of methylthioadenosine phosphorylase (MTAP) was identified, which is an important protein for salvaging adenine and methionine [13]. MTAP is located upstream of CDKN2A, frequently deficient in cancers, and often codeleted with p16 [13].

Also of great note, a nonsynonymous mutation in the E1A binding protein p300 (EP300) was identified. This protein functions as a histone acetyltransferase that regulates transcription via chromatin remodeling and is important in cell proliferation and differentiation [14]. EP300 interacts with hundreds of cellular transcriptional regulators [15] and is a key regulator of p53 function [16]. The observed mutation converts a glutamine to leucine at position 340 within the TAZ1 (CH1) zinc finger domain, which interacts with numerous transcription factors [17] and viral oncoproteins including human papillomavirus E7 [18]. The glutamine that is mutated is known to be involved in the interaction with STAT2, a key component of the interferon response, and potentially many other targets of the TAZ1 domain [19]. EP300 is frequently mutated in several tumour types [20–22] and its inactivation is thought to play a major role in the development of small cell lung cancer [21]. Loss of EP300 function would result in unopposed histone deacetylation, potentially creating an opportunity for targeted therapy with histone deacetylase (HDAC) inhibitors [23].

There was also a deletion identified in PPP1R13B which encodes the apoptosis stimulating of p53 protein 1 (ASPP1) [24]. Specifically, ASPP1 binds to p53 and enhances its ability to specifically stimulate expression of proapoptotic target genes, but not genes involved in cell cycle arrest [25]. Thus, ASPP1 functions as a tumor suppressor gene and has been shown to be downregulated in breast cancer [25] and leukemia cell lines [26], suggesting that the loss of this gene may play an important role in cancer progression. The role of the mutated genes in initiation and progression of AciCC identified in our study will require further investigation.

## 5. Discussion

AciCC is the least aggressive major salivary gland malignancy [27]. Typically these lesions are present at an early stage with low-grade histology and are cured at a high rate solely with surgery [4]. However, a subset presents with higher grade histology and/or advanced local, regional, and distant disease that portends a poorer outcome despite the addition of adjuvant radiation [4]. Currently, there are few treatment options available to be offered these patients when they relapse. For rare tumors such as AciCC, the standard mechanism to identify chemotherapeutic agents through a series of phase I, II, and III trials is not feasible due to limited patient numbers. A focused strategy based on tumor biology is necessary. The advent of massively parallel sequencing has led to incredible advances in the understanding of tumor genetics and biology. Recent exome sequencing of head and neck squamous cell carcinoma (HNSCC) has revealed that the mutational landscape of HNSCC is dominated by mutations in tumor suppressor genes, with only rare targetable mutations in oncogenes [28, 29]. However, studies of other cancers such as melanoma have revealed clearly targetable changes such as activating mutations in BRAF, which have already had a profound impact on clinical care [30]. Our study has provided the first glimpse of the genetic underpinnings of AciCC, highlighting changes in the tumor suppressors CDKN2A and PPP1R13B. There was also a mutation in the histone acetyltransferase EP300 that could reduce the acetylation of various targets. This mutation may make this tumor more susceptible to histone deacetylase inhibitors, which are already showing promise *in vitro* and in early-stage clinical trials [31, 32]. Future large-scale studies of salivary malignancies utilizing next generation sequencing (as for other cancers [33, 34]) will provide hope for improved patient outcomes.

## 6. Conclusion

Acinic cell carcinoma is a relatively rare salivary gland malignancy that typically has a favorable prognosis when treated solely with surgery. A small subset of these cancers present with advanced-stage disease which can be associated with poorer survival despite the addition of adjuvant radiation. Further study is necessary to understand the biology of salivary gland malignancies in order to develop adjuvant therapies to improve outcomes for patients with high-risk disease.

## Supplementary Material

Supplementary Table: Complete list of somatic mutations identified in an acinic cell carcinoma. Single nucleotide polymorphisms (SNPs) were identified in the manner outlined in the bioinformatics methods of Section 3 of the manuscript. Variants were filtered against the gene exclusion list from Fuentes et al. [10].

## Figures and Tables

**Table 1:** Somatic non-synonymous single nucleotide variations.

Gene	Chr	Position	Reference allele	Tumor allele	Zygosity	Region	Type	Transcript	Exon	CDS position	Protein change
**APBA1**	9	72067091	C	T	Hetero	Exonic	Nonsynonymous	NM_001163	9	c.G1915A	p.D639N
**ARHGAP5**	14	32560713	G	T	Hetero	Exonic	Nonsynonymous	NM_001030055	2	c.G838T	p.V280L
**CCDC74A**	2	132288362	T	C	Hetero	Exonic	Nonsynonymous	NM_138770	3	c.T506C	p.M169T
**EP300**	22	41523603	A	T	Hetero	Exonic	Nonsynonymous	NM_001429	4	c.A1019T	p.Q340L
**GRIK3**	1	37324744	G	A	Hetero	Exonic	Nonsynonymous	NM_000831	7	c.C1069T	p.R357C
**KIAA0319L**	1	35972628	G	T	Hetero	Exonic	Stop-gain	NM_024874	3	c.C251A	p.S84X
**KIAA1109**	4	123207860	G	C	Hetero	Exonic	Nonsynonymous	NM_015312	51	c.G9202C	p.D3068H
KRT18	12	53343059	C	A	Hetero	Exonic	Nonsynonymous	NM_199187	2	c.C102A	p.S34R
**LRRC1**	6	53778709	A	G	Hetero	Exonic	Nonsynonymous	NM_018214	11	c.A1048G	p.I350V
**LRRC8E**	19	7963808	G	A	Hetero	Exonic	Nonsynonymous	NM_025061	3	c.G401A	p.S134N
MYO10	5	16694556	G	A	Hetero	Exonic	Nonsynonymous	NM_012334	27	c.C3724T	p.R1242C
**SAMD8**	10	76910566	A	G	Hetero	Exonic	Nonsynonymous	NM_144660	2	c.A280G	p.M94V
SLC25A36	3	140675512	C	A	Hetero	Exonic	Nonsynonymous	NM_018155	2	c.C185A	p.P62H
**WARS2**	1	119575781	T	C	Hetero	Exonic	Nonsynonymous	NM_015836	6	c.A836G	p.H279R

Chr: chromosome, Bolded genes are in cosmic v61.

**Table 2:** Somatic copy-number aberrations.

Gene	Chr	Start coordinate	End coordinate	Adjusted mean log ratio	*P* value	Adjusted *P* value	Gain/loss
**AAK1**	2	69752135	69752264	1.719	1.76*E* − 006	1.54*E* − 002	Gain
**ABCA1**	9	107586742	107586862	1.908	1.50*E* − 007	2.40*E* − 003	Gain
ABCA12	2	215890389	215890509	1.846	7.60*E* − 006	3.83*E* − 002	Gain
**AGAP1**	2	236791980	236792100	2.084	6.34*E* − 009	1.35*E* − 004	Gain
**ALDH1A1**	9	75533626	75533746	1.595	5.94*E* − 006	3.25*E* − 002	Gain
**ASAP2**	2	9508540	9508660	1.931	1.08*E* − 006	1.13*E* − 002	Gain
**CDKN2A**	9	21974440	21974830	−4.943	1.48*E* − 031	1.42*E* − 026	Loss
**CERKL**	2	182430138	182430258	1.672	1.12*E* − 006	1.13*E* − 002	Gain
**CLASP1**	2	122098419	122098539	1.990	2.29*E* − 008	4.38*E* − 004	Gain
**ETV5**	3	185823397	185823517	−1.623	4.52*E* − 006	2.71*E* − 002	Loss
**EXD3**	9	140250649	140250877	1.883	1.22*E* − 005	4.65*E* − 002	Gain
**GABBR2**	9	101156436	101156556	1.864	9.79*E* − 008	1.70*E* − 003	Gain
**GSN**	9	124076166	124076316	1.636	8.28*E* − 006	3.96*E* − 002	Gain
**IL18RAP**	2	103040817	103040937	1.602	6.85*E* − 006	3.64*E* − 002	Gain
**INPP4A**	2	99136444	99136684	1.894	1.99*E* − 006	1.65*E* − 002	Gain
**ITGA4**	2	182388877	182388997	1.965	1.44*E* − 006	1.38*E* − 002	Gain
LOC375190	2	24390449	24390569	2.155	1.82*E* − 007	2.68*E* − 003	Gain
LOC96610	22	22657565	22657685	1.907	1.01*E* − 005	4.29*E* − 002	Gain
**MERTK**	2	112765945	112766169	1.473	7.37*E* − 006	3.81*E* − 002	Gain
**MERTK**	2	112778145	112778265	1.707	9.88*E* − 006	4.29*E* − 002	Gain
**MFSD6**	2	191354482	191354602	1.980	5.64*E* − 007	6.35*E* − 003	Gain
MTAP	9	21837897	21838017	−4.841	4.56*E* − 045	8.72*E* − 040	Loss
**NGEF**	2	233745847	233745967	1.612	8.93*E* − 006	4.17*E* − 002	Gain
**NUP188**	9	131719210	131719330	1.627	2.65*E* − 006	2.03*E* − 002	Gain
POLR1E	9	37489294	37489414	2.029	2.27*E* − 007	3.10*E* − 003	Gain
**POMT2**	14	77753065	77753185	1.796	3.35*E* − 006	2.46*E* − 002	Gain
**PPIG**	2	170460673	170460793	1.944	2.96*E* − 007	3.77*E* − 003	Gain
**PPP1R13B**	14	104201451	104201571	−1.855	3.88*E* − 006	2.48*E* − 002	Loss
**PSMD5**	9	123591371	123591491	1.845	9.39*E* − 006	4.28*E* − 002	Gain
**RNASEH1**	2	3596211	3596331	1.911	5.03*E* − 006	2.83*E* − 002	Gain
**SH3RF3**	2	109988029	109988149	2.909	2.23*E* − 013	7.12*E* − 009	Gain
**SLC46A2**	9	115642010	115642130	1.621	1.14*E* − 005	4.45*E* − 002	Gain
**SULT6B1**	2	37415566	37415686	1.530	1.04*E* − 005	4.32*E* − 002	Gain
**TESK1**	9	35607906	35608026	1.688	4.54*E* − 006	2.71*E* − 002	Gain
**TTC27**	2	32929895	32930015	1.466	1.09*E* − 005	4.43*E* − 002	Gain

Chr: chromosome, bolded genes are in cosmic v61.
